# Transcriptomics of the Vaccine Immune Response: Priming With Adjuvant Modulates Recall Innate Responses After Boosting

**DOI:** 10.3389/fimmu.2018.01248

**Published:** 2018-06-05

**Authors:** Francesco Santoro, Elena Pettini, Dmitri Kazmin, Annalisa Ciabattini, Fabio Fiorino, Gregor D. Gilfillan, Ida M. Evenroed, Peter Andersen, Gianni Pozzi, Donata Medaglini

**Affiliations:** ^1^Laboratorio di Microbiologia Molecolare e Biotecnologia (LA.M.M.B.), Dipartimento di Biotecnologie Mediche, Università di Siena, Siena, Italy; ^2^Emory Vaccine Center, Emory University, Atlanta, GA, United States; ^3^Department of Medical Genetics, Oslo University Hospital, University of Oslo, Oslo, Norway; ^4^Department of Infectious Disease Immunology, Statens Serum Institut, Copenhagen, Denmark

**Keywords:** vaccine adjuvant, RNA sequencing, adaptive immunity, recall innate response, CAF01

## Abstract

Transcriptomic profiling of the immune response induced by vaccine adjuvants is of critical importance for the rational design of vaccination strategies. In this study, transcriptomics was employed to profile the effect of the vaccine adjuvant used for priming on the immune response following re-exposure to the vaccine antigen alone. Mice were primed with the chimeric vaccine antigen H56 of *Mycobacterium tuberculosis* administered alone or with the CAF01 adjuvant and boosted with the antigen alone. mRNA sequencing was performed on blood samples collected 1, 2, and 7 days after priming and after boosting. Gene expression analysis at day 2 after priming showed that the CAF01 adjuvanted vaccine induced a stronger upregulation of the innate immunity modules compared with the unadjuvanted formulation. The immunostimulant effect of the CAF01 adjuvant, used in the primary immunization, was clearly seen after a booster immunization with a low dose of antigen alone. One day after boost, we observed a strong upregulation of multiple genes in blood of mice primed with H56 + CAF01 compared with mice primed with the H56 alone. In particular, blood transcription modules related to innate immune response, such as monocyte and neutrophil recruitment, activation of antigen-presenting cells, and interferon response were activated. Seven days after boost, differential expression of innate response genes faded while a moderate differential expression of T cell activation modules was appreciable. Indeed, immunological analysis showed a higher frequency of H56-specific CD4+ T cells and germinal center B cells in draining lymph nodes, a strong H56-specific humoral response and a higher frequency of antibody-secreting cells in spleen of mice primed with H56 + CAF01. Taken together, these data indicate that the adjuvant used for priming strongly reprograms the immune response that, upon boosting, results in a stronger recall innate response essential for shaping the downstream adaptive response.

## Introduction

Vaccines based on purified antigens are often poorly immunogenic and need to be formulated with adjuvants or delivery systems, to increase the amount, quality, and duration of the immune response to vaccination as well as to ensure long-lived immunological memory and protection (1). Adjuvants are substances capable of enhancing and properly skewing the immune responses to the vaccine antigen, and their choice can dramatically affect the type and the magnitude of the adaptive immune response to the vaccine antigen, by impacting on the innate response starting signal (2).

Profiling the mode of action of different adjuvants is of critical importance for the rational design of vaccination strategies, based on heterologous combinations of vaccine formulations for priming and boosting, and for predicting the protective potential of an adjuvant for a given vaccine antigen (3–8). The immune profile and efficacy of five different adjuvants, combined with vaccine antigens from *Mycobacterium tuberculosis*, influenza, and *Chlamydia*, were tested in murine infection models within the ADITEC project on advanced immunization technologies (6, 9). We have also characterized the antigen-specific T and B cell responses after both parenteral and mucosal priming with vaccine formulations including different adjuvants or delivery systems, and combining heterologous prime-boost schedules, demonstrating the crucial role of both the immunization route and vaccine composition (3, 5, 10–13).

A systems biology approach, integrating gene expression data with immunological results, has been used to study the human response to different vaccines (14–26). Systems biology is an interdisciplinary approach to analyze multiple data types related to complex biological interactions by using computational analysis and mathematical modeling. The systems biology approach was first applied to characterize the immune response elicited by the yellow fever vaccine YF-17D in humans (23) and, more recently, to a variety of other vaccines including the study of adjuvanted and non-adjuvanted influenza vaccines in adults (21) and children (22, 27).

This approach has been recently applied to investigate the priming properties of different vaccine adjuvants at early time points after priming in the mouse model, using genome wide microarrays, and identified shared blood gene modules enriched in T follicular helper and germinal center responses (28). Mathematical models have been also developed to predict the probability of antigen-specific CD4+ T cells to expand and disseminate *in vivo* into other secondary lymphoid organs following primary immunization with adjuvanted vaccine formulations (29, 30).

CAF01 is a promising vaccine adjuvant that has been tested in five phase I clinical trials, administered in combination with four different antigens including the H56 tuberculosis (TB) vaccine antigen (Clinical trial no. NCT00922363), to evaluate its safety, tolerability, and immunogenicity. CAF01 is a liposomal adjuvant system composed of cationic liposome vesicles [dimethyldioctadecylammonium (DDA)] combined with a synthetic variant of cord factor of the mycobacterial cell wall [trehalose 6,6-dibehenate (TDB)], which was shown to activate macrophages and dendritic cells (DCs) through a specific innate activation program *via* Syk–Card9–Bcl10–Malt1 (31). CAF01 promotes vaccine depot formation, prolonging the release of antigens and stimulating the induction of T follicular helper cells into the draining lymph nodes (dLN), together with combined Th1 and Th17 responses and the generation of a robust, long-lived memory response in mice (32–34). CAF01 has been used as a component of a promising TB vaccine candidate in combination with the chimeric antigen H56 of *M. tuberculosis* consisting of the antigen Ag85B fused to the 6-kDa early secretory antigenic target and the latency-associated protein Rv2660c (35, 36). The phase I clinical trials showed that CAF01 is safe and induces a strong cell-mediated immune response in addition to antibodies in humans (37, 38).

In this work, we have analyzed, through a systems biology approach, how the CAF01 adjuvant, combined with the H56 antigen used for priming, programs the immune response to downstream re-exposure to the same antigen. Mice were primed with H56 + CAF01 or H56 alone and boosted with the H56 antigen. A very low antigen dose was used for the boost to select antigen-specific clones of T and B cells and to mimic the challenge with the pathogen. RNA sequencing was used to characterize the blood transcriptome at several time points (1, 2, and 7 days) both after priming and after boosting, allowing us to follow the transcriptomic profile of the same animals over time. Transcriptomic data were analyzed together with immunological data on both cellular (antigen-specific CD4+ T cells and germinal center B cells in dLN) and humoral responses (quantification of H56-specific IgG up to 7 weeks after boost). These studies characterize, for the first time, using a systems biology approach, the modulation of the response elicited after prime-boost vaccination with the CAF01 adjuvant.

## Materials and Methods

### Mice

Seven-week-old female C57BL/6 mice, purchased from Charles River (Lecco, Italy), were housed under specific pathogen-free conditions in the animal facility of the Laboratory of Molecular Microbiology and Biotechnology (LA.M.M.B.), Department of Medical Biotechnologies at University of Siena, and treated according to national guidelines (Decreto Legislativo 26/2014). All animal studies were approved by the Italian Ministry of Health with authorization no. 1004/2015-PR on September 22 2015.

### Experimental Design

**Figure 1:**
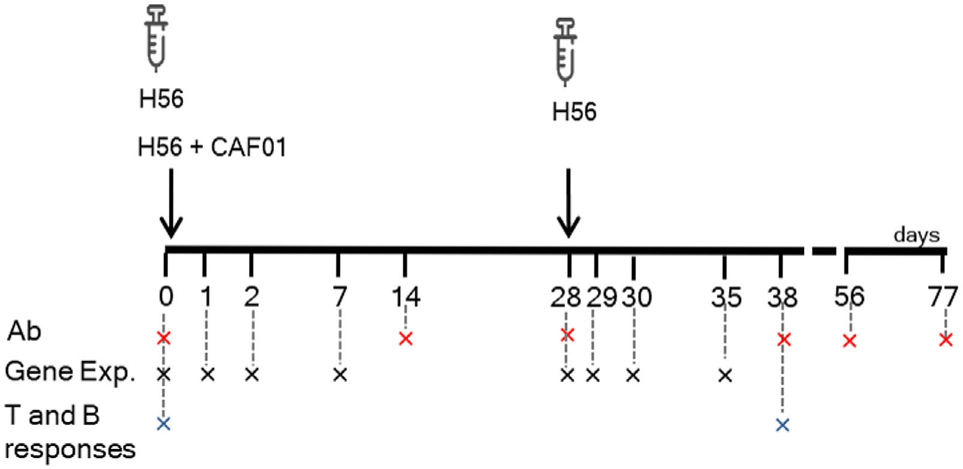
Study design and samples collection. C57BL/6 mice were subcutaneously primed with H56 antigen alone or combined with CAF01, and boosted at day 28 with H56. Blood samples were collected at days 0, 14, 28, 38, 56, and 77 for antibody analysis (Ab) and at days 0, 1, 2, 7, 29, 30, and 35 (corresponding to days 1, 2, and 7 after boosting) for gene expression (Gene Exp). dLN and spleens were collected at days 0 and 38 (10 days after boosting) to evaluate the secondary CD4+ T and B cell responses.

Mice were subcutaneously primed with the chimeric TB vaccine antigen H56 administered alone or in combination with the liposome system CAF01, and boosted after 4 weeks with H56 antigen alone (Figure 1). Both the innate and adaptive immune responses elicited by the two vaccine formulations were explored. T and B cells responses were characterized 10 days after boosting (day 38) within the local dLN and spleen, while the induction of H56-specific IgG serum response was followed at different time points, up to 7 weeks post boost (day 77). In parallel, other groups of mice were immunized with the same vaccine schedule described earlier, and the molecular signature of the vaccine formulations was explored by sequencing RNA from blood at early time points (1, 2, and 7 days) after primary and secondary immunizations, and thus following the transcriptomic profile over time without sacrificing the animal (Figure 1).

### Immunizations

Groups of four to five mice were immunized by the subcutaneous route at the base of the tail. Vaccine formulations consisted of the chimeric TB vaccine antigen H56 (2 μg/mouse for priming, 0.5 μg/mouse for boosting; Statens Serum Institut, Denmark), administered alone or combined with the adjuvant CAF01 (250 µg DDA and 50 µg TDB) per mouse (Statens Serum Institut, Denmark). Vaccine formulations containing CAF01 were injected in a volume of 150 μl/mouse of 10 mM Tris buffer, while formulations containing H56 alone in a volume of 100 μl/mouse of PBS. Mice primed with both H56 + CAF01 or H56 alone were boosted with H56 antigen 4 weeks later.

### Blood Sample Collection

For antibody analysis, blood samples were taken by temporal plexus bleed on days 0, 14, 28, 38, 56, and 77 after primary immunization. Blood was incubated for 30 min at 37°C, centrifuged at 1,200 × *g* at 4°C for 15 min, and then serum was collected and stored at −80°C until further analysis.

For transcriptomic analysis, blood samples were collected from individual mice on days 1, 2, 7, 28, 29, 30, and 35 after priming. Blood (200 µl) was mixed in a 1:2.8 ratio with PaxGene reagent, in a 2 ml Biosphere Eppendorf tube (39, 40). The tube was inverted five times and incubated at room temperature for 2 h before freezing at −80°C. Total RNA was extracted using the PaxGene blood RNA kit (Qiagen, Germany) following manufacturer’s instructions and resuspended in 60 µl of BR5 buffer. RNA was quantified using (i) the total RNA nano kit on a 2100 Bioanalyzer (Agilent Technologies, USA), (ii) the RNA broad range kit on a Qubit 2.0 (Thermo Fisher, USA), and (iii) the Nanophotometer (Implen, Germany).

### Illumina Sequencing

Libraries were prepared using TruSeq stranded RNA reagents (Illumina, USA) according to manufacturer’s instructions, using 500 ng of total RNA input per sample with dual indexing to enable all libraries to be sequenced in the same run. Pooled libraries were sequenced on four runs of an Illumina NextSeq 500 instrument with 75 bp single end reads. Image analysis and base calling were performed using Illumina’s RTA software version 2.4.11 and bcl2fastq version 2.18.0.12. Reads were filtered to remove those with low base call quality using Illumina’s default chastity criteria. Raw sequence data were deposited in Sequence Read Archive under Bioproject number PRJNA437839.

### Multiparametric Flow Cytometric Analysis

B and T responses were analyzed in dLN (sub iliac, medial, and external). Samples were mashed onto 70 µm nylon screens (Sefar Italia, Italy) and washed two times in complete RPMI medium (cRPMI, Lonza, Belgium) containing with 100 U/ml penicillin/streptomycin and 10% fetal bovine serum (Gibco, USA). Samples were treated with red blood cells lysis buffer according to manufacturer instruction (eBioscience, USA). Cells were incubated for 30 min at 4°C in Fc-blocking solution [cRPMI medium with 5 µg/ml of CD16/CD32 mAb (clone 93; eBioscience, USA)]. To evaluate germinal center B lymphocytes from dLN, cells were stained with AF700-conjugated anti-CD45R (anti-B220, clone RA3-6B2; BD Biosciences), BV421-conjugated anti-GL7 (clone GL7; BD Biosciences), and PE-Cy7-conjugated anti-CD95 (clone Jo2; eBioscience). To evaluate H56-specific CD4+ T lymphocytes, cells from dLN were stained for 1 h at RT with PE-conjugated I-A(b) *M. tuberculosis* Ag85B precursor 280–294 (FQDAYNAAGGHNAVF) tetramer (kindly provided by NIH MHC Tetramer Core Facility, Emory University, Atlanta, GA, USA), washed and surface stained with HV500-conjugated anti-CD4 (clone RM4-5; BD Biosciences), and BV786-conjugated anti-CD44 (clone IM-7; BD Biosciences). Samples were labeled with Live/Dead Fixable Near-IR Stain Kit according to manufacturer’s instructions (Invitrogen, USA). All antibodies and tetramers were titrated for optimal dilution. Approximately 5–10 × 10^5^ cells were acquired on a LSRFortessa_TM_ X-20 flow cytometer (BD Biosciences). Data analysis was performed using FlowJo v10 (TreeStar, USA).

### IgG ELISpot

Antibody-secreting cells (ASC) were evaluated in the spleen by ELISpot PLUS for mouse IgG kit (Mabtech) 10 days post boost. Multiscreen filter (PVDF) plates (Millipore, USA) were pre-wet for 2 min with EtOH 70%, washed with sterile dH_2_O and coated with H56 (5 µg/ml) diluted in PBS (Sigma-Aldrich) for the detection of antigen-specific IgG. After incubation overnight at 4°C, the coated wells were washed with sterile PBS and blocked for 30 min with serum free CTL-Test B culture medium (CTL, USA) supplemented with 1% l-glutamine (Sigma-Aldrich, USA). After removal of the blocking medium, 1 × 10^6^ cells/well were added in a volume of 100 µl of CTL-Test B medium for the analysis of H56-specific IgG ASC. Each sample was assayed in triplicate, and the plates were incubated overnight for 20 h at 37°C in a humidified atmosphere with 5% CO_2_. After incubation, cells were removed by washing with PBS and 100 µl/well of anti-IgG biotinylated detection antibody (Mabtech, Sweden), diluted to 1 µg/ml in PBS containing 0.5% fetal calf serum (FCS, Gibco), was added. After incubation for 2 h at room temperature followed by washing steps, ELISpot plates were incubated with 50 µl/well of streptavidin–horseradish peroxidase (Mabtech) diluted 1:1,000 for 1 h at room temperature. Plates were washed again with PBS, and then 100 µl/well TMB substrate solution (Mabtech) was added for approximately 10 min. The reaction was stopped by extensive washing in dH_2_O, and plates were then dried in the dark at room temperature. The number of spots was determined by plate scanning and analysis services performed at Cellular Technology Limited (Germany).

### Enzyme-Linked Immunosorbent Assay (ELISA)

Serum H56-specific IgG were quantified by ELISA at different time points. Flat bottomed Maxisorp microtiter plates (Nunc, Denmark) were coated with H56 (0.5 µg/ml) for 3 h at 37°C and overnight at 4°C in a volume of 100 μl/well. Plates were washed and blocked with 200 µl/well of PBS containing 1% BSA (Sigma-Aldrich) for 2 h at 37°C. Serum samples were added (100 μl/well) and titrated in twofold dilution in duplicate in PBS supplemented with 0.05% Tween 20 and 0.1% BSA (diluent buffer). After incubation for 2 h at 37°C, samples were incubated with the alkaline phosphatase-conjugate goat anti-mouse IgG (100 μl/well diluted 1:1,000 in diluent buffer, Southern Biotechnology, USA) for 2 h at 37°C and developed by adding 200 μl/well of 1 mg/ml of alkaline phosphatase substrate (Sigma-Aldrich). The optical density was recorded using a Multiskan FC Microplate Photometer (Thermo Scientific). Antibody titers were expressed as the reciprocal of the highest dilution with an OD value ≥ 0.2 after background subtraction.

### Bioinformatic Analysis

Raw reads were aligned to mouse genome (UCSD mm10 annotation), and counts were generated using STAR (41). Differential gene expression was determined using edgeR (42) with the generalized linear model (GLM) fitting approach for both longitudinal analysis and pairwise adjuvanted *versus* non-adjuvanted vaccine comparisons at each time point. For each comparison, edgeR generated a list of genes associated with a log fold-change and to a false discovery rate (FDR, i.e., the *P* value after multiple test correction). Genes were considered significantly differentially expressed when FDR was <0.05. For enrichment analysis, lists of genes were sorted by FDR, then mouse gene identifiers were converted to human gene identifiers using an in house script utilizing the BiomaRt Bioconductor package. Enrichment analysis was performed using the tmod R package (43) with blood transcription modules (BTMs) developed by Li and coworkers (19). Significance of module enrichment was assessed using the CERNO statistical test (a modification of Fisher’s combined probability test) and corrected for multiple testing using the Benjamini–Hochberg correction.

### Statistical Analysis

Analysis was performed on individual samples, and data were reported in box plots encompassing minimum and maximum values. The Kruskal–Wallis test, followed by Dunn’s post test for multiple comparison, was used to assess statistical differences among the number of (i) Ag85B-specific CD4+ T cells, (ii) germinal center B cells, and (iii) H56-specific ASC in different groups of mice (naïve, primed with antigen H56 alone or primed with H56 + CAF01). H56-specific IgG were reported in graphs as log-transformed geometric mean titers with 95% confidence intervals and compared using *t*-test. Statistical significance was defined as *P* < 0.05, and analyses were performed using Graph Pad Prism version 7 (GraphPad Software, USA).

## Results

### Antigen-Specific CD4+ T-Cell Response

**Figure 2:**
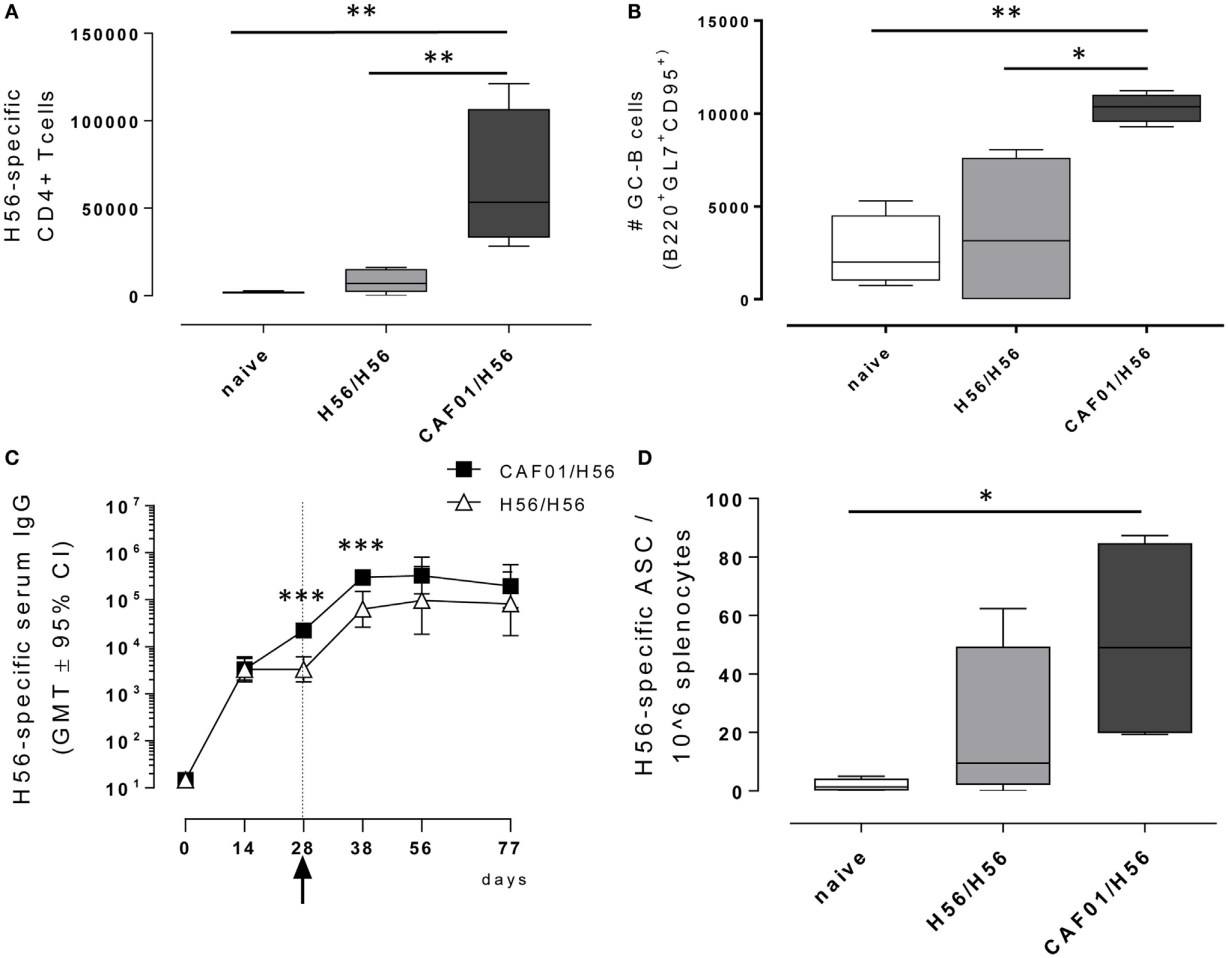
Adaptive immune response following booster immunization. C57BL/6 mice were subcutaneously immunized as reported in Figure 1. dLN were collected 10 days after booster immunization and analyzed for the T and B recall responses. **(A)** Number of Ag-specific CD4+ T cells, identified as CD4+ CD44+ tetramer Ag85B-specific cells. Mean values ± SEM of five mice per group are reported. **(B)** Number of germinal center B cells, identified as GL7+ CD95+ among B220+ B cells in dLN. **(C)** H56-specific serum IgG titers determined on days 0, 14, 28, 38, 56, and 77 following priming, by ELISA. Antibody titers values are reported as GMT ± 95% CI of five mice per group. Arrow indicates day 28 that is the time of boosting. **(D)** H56-specific antibody-secreting cells (ASC) detected in spleens by ELISPOT assay. Kruskal–Wallis test was used to assess statistical differences between groups of mice primed with H56 + CAF01 *versus* H56 alone (*P ≤ 0.05, **P ≤ 0.01, and ***P ≤ 0.001).

Antigen-specific CD4+ T cell responses were analyzed in mice primed with the chimeric TB vaccine antigen H56 administered alone or in combination with the liposome system CAF01, and boosted after 4 weeks with H56 antigen alone (Figure 1). T cell responses were characterized 10 days after boosting (day 38) within the local dLN. CD4+ T cells specific for the immunodominant epitope Ag85B, part of the H56 fusion protein, were identified using Ag85B_280–294_-complexed MHC class II tetramers. Staining specificity of Ag85B_280–294_-complexed MHC class II tetramers was determined using a control tetramer complexed with an unrelated antigen, which showed a level of staining below 0.02% (data not shown). Ten days after booster immunization with H56 antigen, the number of tetramer-binding CD4+ T cells in dLN was about 66,000 (±39,000) in mice primed with H56 + CAF01. This was significantly higher than the amount of antigen-specific CD4+ T cells present in mice primed with the H56 antigen alone (8,260 ± 6,000, *P* < 0.01, Figure 2A). The percentage of antigen-specific CD4+ T cells in respect to total activated CD4+ T cells was 0.55% and 0.07% in mice primed with H56 + CAF01 and H56 antigen alone, respectively (*P* < 0.05, data not shown). These data show that inclusion of CAF01 in the priming formulation induces an antigen-specific proliferation of CD4+ T cells which is not observed with the antigen alone.

### Characterization of the B-Cell Response

B cells responses were characterized 10 days after boosting (day 38) within the local dLN and spleen. Similar to the CD4+ T recall response, the reactivation of the B cell response was significantly higher in mice that had been primed with H56 + CAF01 compared with H56-primed mice (Figure 2B). Indeed, a significant expansion of the B220+ GL7+ CD95+ germinal center B cells (GC-B cells) 10 days following booster immunization was measured in mice primed with the CAF01 adjuvant (*P* < 0.05 *versus* H56-primed mice, *P* < 0.01 *versus* naïve mice), while the amount of cells detected in mice primed with H56 alone was not significantly higher compared with naïve mice (Figure 2B). The observed GC-B cells response is clearly a recall response of cells elicited by the primary immunization, since we have previously demonstrated that primary immunization with H56 alone does not stimulate the germinal center reaction, while the CAF01 adjuvant is a strong promoter of this reaction (4).

The induction of antigen-specific IgG antibody responses was also assessed at different time points after priming and boosting (Figure 2C). The IgG response elicited by both H56 alone or combined with CAF01 was very similar at 12 days after priming, while a significantly higher response was observed at day 28 and maintained 10 days after boosting in mice primed with antigen and adjuvant compared with animals primed with antigen alone (day 38, *P* < 0.001). This was further confirmed by assessing antigen-specific ASC in the spleen collected 10 days after booster immunization (Figure 2D). The number of H56-specific ASC was higher in mice primed with H56 + CAF01 compared with animals primed with H56 alone with mean values of 51 and 20, respectively.

### Blood Gene Expression Compared With Pre-Immunization and Pre-Boost Baselines

The molecular signature of the vaccine formulations was analyzed in mice primed with H56 alone or in combination with CAF01, and boosted with H56 antigen alone (Figure 1). The transcriptomic profile was followed over time (without sacrificing the animals), sequencing blood RNA samples collected at early time points (1, 2, and 7 days) after primary and secondary immunizations (4 samples/each time point). An average of 7.2 µg total RNA/sample (range 1.473–60.552 μg/sample, as quantified by fluorimetry) was obtained. The isolated RNA was generally of high quality, with an average RNA Integrity number of 9.05 (range 5.8–9.9), as measured by the Bioanalyzer, and a mean 260/280 absorbance ratio of 2.17.

**Figure 3:**
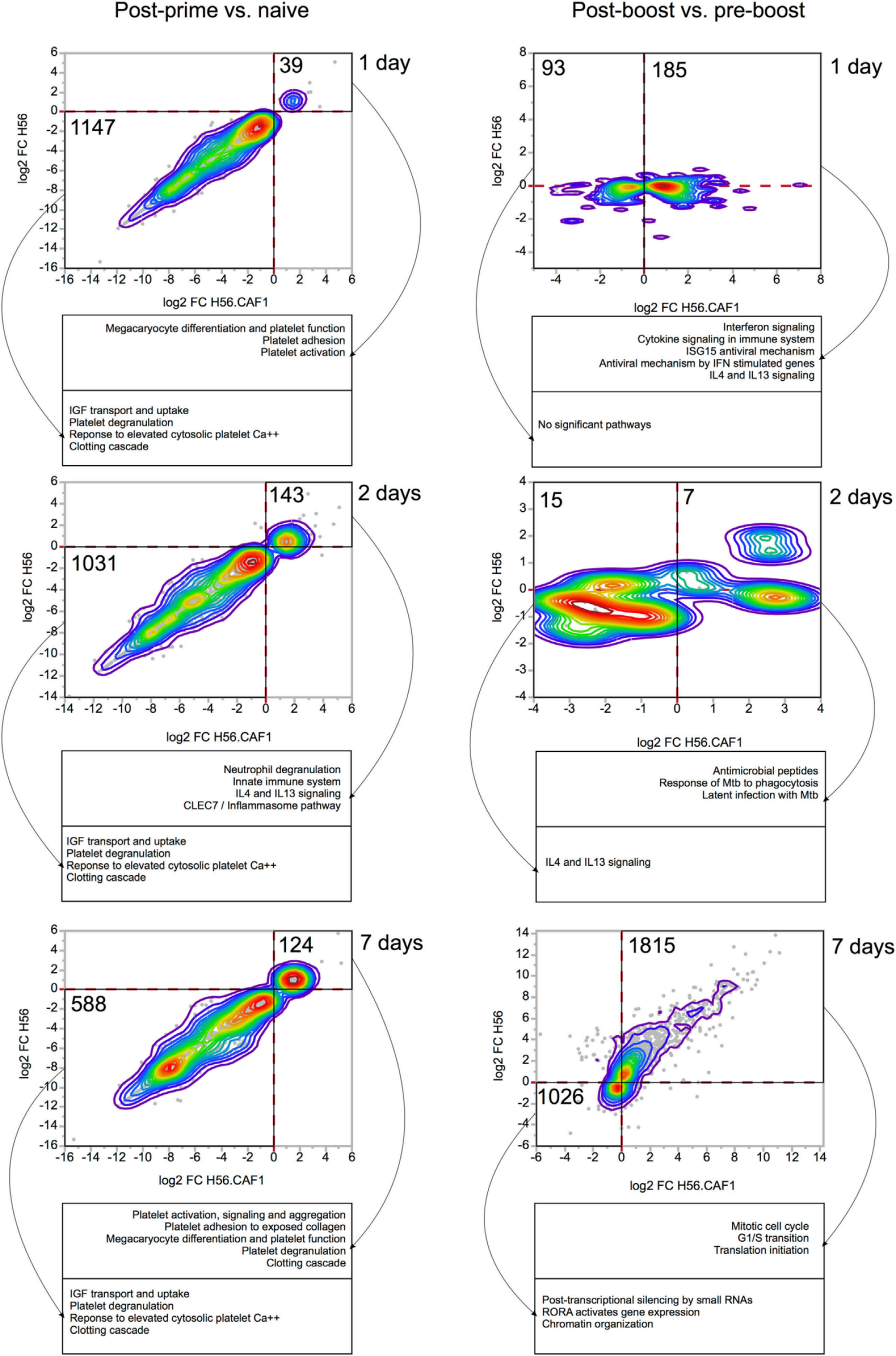
Comparisons of gene expression changes elicited by H56 and H56 + CAF01 vaccine formulations. Each bivariate plot illustrates the comparison at one time point, as indicated. For the post-prime time points, the comparisons are represented relative to the naïve baseline. For post-boost time points, the baseline is the pre-boost time point. Axes represent log 2 of baseline-normalized expression values. Each dot represents a gene, and colored lines indicate the density of dots on the graph. Only genes significantly regulated (FDR < 0.05) by at least one vaccine were included and plotted. Numbers indicate the number of genes included in areas indicated by solid black lines. Dashed red lines indicate 0 values (no change in gene expression). Functional analysis was done using Reactome database, and representative significantly enriched pathways are included in captions under each plot.

Gene expression in the blood of mice primed either with H56 + CAF01 or H56 alone was first compared with gene expression in blood collected before immunization. In Figure 3 the left column reports bivariate plots showing differences in gene expression between the H56 + CAF01 and H56 alone immunized mice at each time point. Differentially expressed genes were subjected to functional analysis using the Reactome database.^1^

At day 1 after priming, 39 genes were found to be upregulated, while 1,147 genes were downregulated by both formulations. The gene modulation was essentially limited to pathways of platelet function adhesion, activation and degranulation, and clotting, as expected for injection and repeated bleeding of mice. At day 2 after priming, 143 genes were upregulated, while 1,031 genes were downregulated. Among the upregulated genes there was an activation of pathways related to innate immunity, neutrophils degranulation and IL4-IL13 signaling. At day 7, 124 genes were upregulated, and 588 were downregulated by both vaccines. Overall, the transcriptomic response was very consistent between the two vaccine formulations.

Four weeks after priming, both groups of mice received a booster immunization with H56 antigen alone. The day before booster immunization, blood was collected from both groups of mice, and gene expression analysis performed at this time point was then used as a baseline. Gene expression in the blood of mice primed with H56 + CAF01 or H56 alone and boosted with H56 was indeed compared with gene expression in blood collected in the same group of mice before booster immunization. In the right column of Figure 3 bivariate plots show differential gene expression at 1, 2, and 7 days after H56 boosting.

Among the genes upregulated 1 day after H56 boost in mice primed with H56 + CAF01, we observed an over representation of genes relevant to innate immune response, centered primarily on the interferon response, as well as IL4 and IL13 signaling. Importantly, these genes were regulated almost exclusively in the H56 + CAF01 primed group, but not in the group that received H56 alone as priming. Two days after boost, only a few genes were found to be differentially expressed, and, again, as at day 1 post boost, gene regulation was observed primarily in the H56 + CAF01 primed group, but not in the group of mice primed with the antigen alone. Interestingly, according to the Reactome analysis, the upregulated genes were enriched in *M. tuberculosis* response genes; however, due to the very small number of genes regulated at this time point, these results should be taken with caution. By 7 days after the boost, the two groups displayed a similar gene expression pattern, mainly related to genes involved in cell cycle and proliferation, suggesting the activation of an adaptive response and clonal expansion of responding antigen-specific cells (Figure 3, right column).

### Effect of CAF01 Adjuvant on Gene Expression

**Figure 4:**
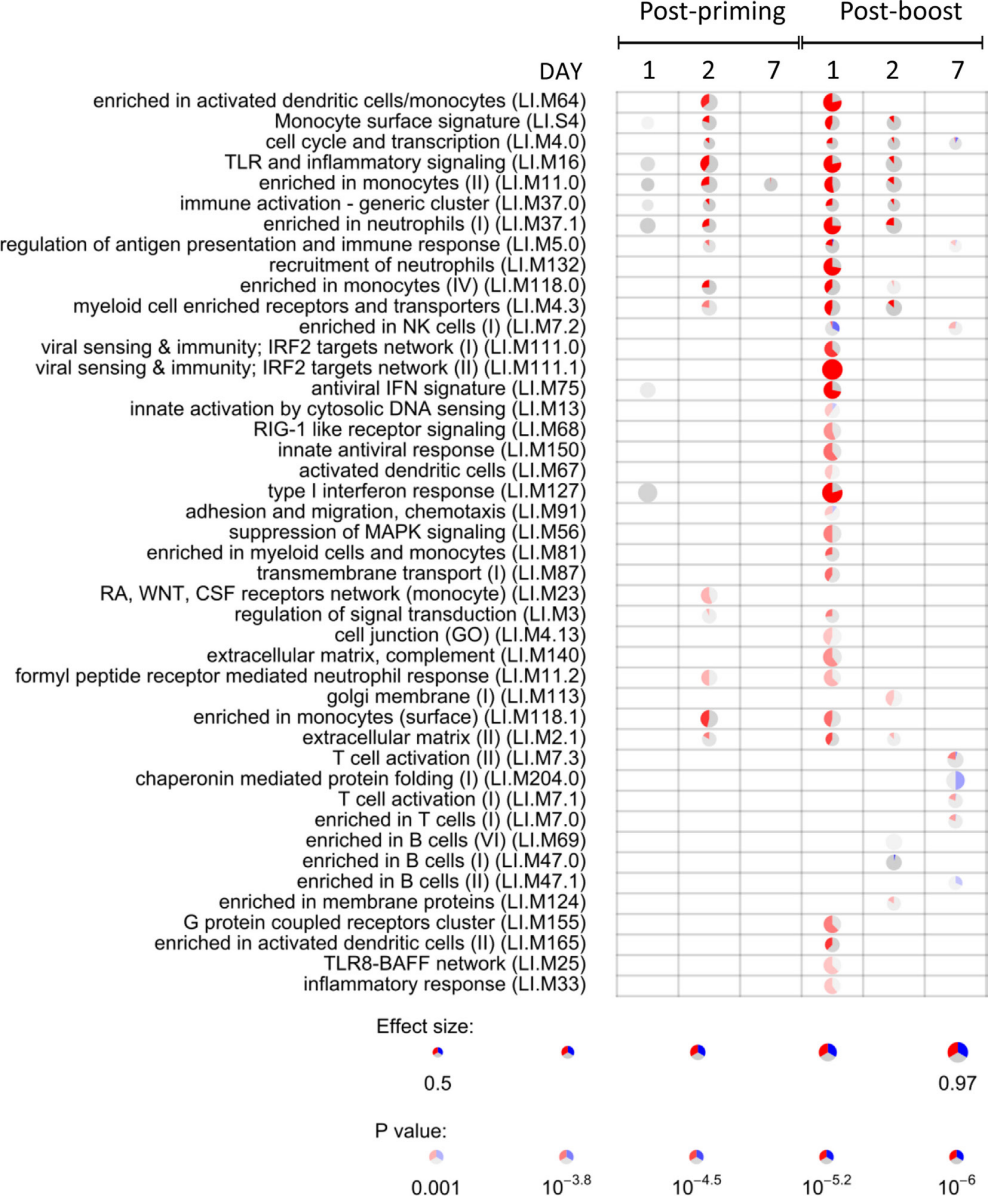
Activation of blood transcription modules (BTMs) by the CAF01 adjuvant. Each column represents a pairwise comparison between blood RNA samples from mice primed with H56 + CAF01 *versus* H56 alone. Both groups were boosted after 4 weeks with H56 alone. Blood samples were collected 1, 2, and 7 days after priming and after boosting. Activation of modules was tested using the FDR-ranked lists of genes generated by edgeR generalized linear model fitting and applying the CERNO test. Rows indicate different BTMs, which were significantly (FDR < 0.001) activated in at least one time point. Each module is represented by a pie in which the proportion of significantly upregulated and downregulated genes is shown in red and blue, respectively. The gray portion of the pie represents genes that are not significantly differentially regulated. The significance of module activation is proportional to the intensity of the pie, while the effect size (area under the curve) is proportional to its size.

To identify the CAF01-specific transcriptomic response, the blood gene expression in mice primed with H56 + CAF01 was compared with that of mice primed with H56 alone. All comparisons were performed at 1, 2, and 7 days after priming and after H56 boost. Table 1 reports the number of differentially expressed genes identified in edgeR by the genewise Negative Binomial Generalized Linear Models algorithm. Lists of genes, sorted by FDR, were converted to human IDs and tested for the significance in the enrichment of BTMs (19). BTMs include 346 sets of genes which are coordinately expressed and exert a specific function, described by the module title, including innate and adaptive immunity or general biological processes such as cell cycle, transcription or signal transduction. Figure 4 reports modules that were found to be significantly enriched (FDR < 0.001) in at least one tested time point. Data analysis at days 1, 2, and 7 after priming indicated that the major differences between the two vaccine formulations were detected at day 2, with 2.2% of differentially expressed genes and upregulation of modules related to innate immunity by the CAF01 adjuvanted vaccine formulation. In particular, modules related to antigen-presenting cells (monocytes and DCs) were found to be enriched, together with neutrophils and TLR inflammatory signaling. Differential gene expression analysis found no significant difference between H56 + CAF01 and H56 alone vaccinated groups at day 1, and only 17 significant genes at day 7 (Table 1). Nevertheless, module enrichment analysis identified seven enriched modules at day 1 and one module at day 7. These modules were also related to innate immune response, indicating that CAF01 modulates the transcriptomic response also at those time points.

**Table 1:** Number of differentially expressed genes in CAF01 adjuvanted mice.

	H56 + CAF01 *versus* H56	Upregulated genes	Downregulated genes	DE genes (%)	Unaffected genes
Post-priming	Day 1	0	0	0 (0)	12,340
	Day 2	191	83	274 (2.2)	12,066
	Day 7	7	10	17 (0.1)	12,323
Post-boosting	Day 1	827	168	995 (8.1)	11,345
	Day 2	134	59	193 (1.6)	12,147
	Day 7	269	490	759 (6.2)	11,581

The most significant differences between the two vaccine formulations could be appreciated after antigen boost, thus demonstrating the key role of the boost in highlighting the skewing of the immune system induced by the adjuvant used for priming. In fact, at day 1 after boost, 34 out of the 44 significant modules were modulated by CAF01, with 8.1% of genes being differentially expressed (Figure 4; Table 1). In particular, the innate immunity compartment was found to be affected: 10 modules regarding monocytes and antigen presentation, three modules associated with neutrophil recruitment and six modules related to interferon response were upregulated, while one module associated with natural killer (NK) cells was downregulated. No enrichment of modules related to B- and T-cell populations was observed at this early time point. At day 2 after boost, the overall difference between the two formulations was lower: 13 modules were significantly enriched, mainly related to innate response with a minor modulation of B cells. Seven days after the boost, despite the relatively high number of differentially expressed genes (Table 1), only eight modules were affected (Figure 4). In particular, these were regarding adaptive immune response with three upregulated modules regarding T cell activation and one downregulated relevant to B cells activities.

## Discussion

Adjuvants have been extensively employed in vaccinology to enhance the immune responses to the antigen and promote the strength and persistence of the resulting immunity. Transcriptomic profiling of the immune response induced by vaccine adjuvants can contribute understanding the mechanism of action of adjuvants and guide the rational design of prime-boost vaccination strategies. A systems biology approach has been recently applied to profile priming properties of different vaccine adjuvants in a preclinical model using genome wide microarrays (24) and to analyze the response to adjuvanted and unadjuvanted influenza vaccines in human studies (18, 19). A clinical study performed on young children vaccinated with TIV vaccine administered with or without MF-59 adjuvant demonstrated that the inclusion of the oil-in-water adjuvant results in a stronger transcriptional response at the early time point post vaccination, including a higher interferon response, and higher resulting HAI titers (22). However, little is understood about the way adjuvants program the immune system for the response to re-exposure to the antigen.

In this study, we sought to investigate how the inclusion of the adjuvant in the vaccine formulation modifies the way the immune system responds to the same antigen, administered at a later time without the adjuvant, thus modeling the re-exposure scenario. We used mRNA sequencing to profile the mice immune response to the vaccine adjuvant CAF01 used in a primary immunization followed by a booster immunization with a low dose of H56 (0.5 μg/mouse) to select antigen-specific clones of T and B cells. While we found almost no difference in the transcriptomic profile after priming with or without the CAF01 adjuvant, strikingly, the effect of the CAF01 adjuvant, used in the primary immunization, could be readily appreciated after a booster immunization with the vaccine antigen alone. Indeed, we observed a significantly stronger upregulation of multiple genes and modules related to the innate immune response following boost in mice that received the adjuvant with the primary immunization compared with the mice that did not. This indicates the potent reprogramming of the immune response to re-exposure by the adjuvant included with the primary immunization. Transcriptomics of the vaccine immune response highlights therefore that priming with adjuvant modulates recall innate responses after boosting. Recent studies have reported the capacity of innate immune cells such as NK cells, monocytes and macrophages to mount a differential immune response upon a secondary contact with the same or distinct stimuli (44, 45). This concept of “memory” related to cells of the innate immune system is revolutionizing our knowledge of the immune system, and could represent an important goal for future vaccination strategies, based on the interaction between adjuvants and innate cells. Our analysis, performed on the whole blood level, does not permit us to dissect the underlying mechanisms of the observed differential response, nevertheless, a new study that will include sorting of the innate cell subpopulations and transcriptional profiling of the isolated populations, is currently in the development phase.

In this study, we extracted total RNA from small volumes of whole blood to make a longitudinal analysis of the transcriptomic response in the same animals bled multiple times during the course of the experiment. This study design and technical approach has been previously employed only in few studies (39, 40) and has proved the benefit of longitudinal observation within the same animal allowing analysis of gene modulation over time. An unexpected limitation introduced by this approach related to the fact that transcripts that we found to be regulated in response to the primary immunization were strongly enriched in genes relevant to platelet activation and aggregation, suggesting that the response to wounding may have masked the vaccine-specific response. Nevertheless we identified day 2 after priming and day 1 after boost as optimal time points to analyze the gene modulation induced by priming with CAF01 adjuvant compared with unadjuvanted vaccine formulation.

While we observed only a moderate differential expression of T cell activation modules 7 days after boost, the immunological analysis performed 10 days after boosting showed a higher frequency of H56-specific CD4+ T cells and germinal center B cells in dLN, a strong H56-specific humoral response and a higher frequency of ASC in spleen of mice primed with H56 + CAF01. We and others have previously shown that the presence of the CAF01 adjuvant combined with the antigen in the vaccine formulation used for priming is crucial for the induction of the germinal center reaction, assessed by the presence of the T follicular and germinal center B cells (4, 46), as well as for a strong T cell mediated immune response with a Th1 and Th17 profile (4, 6, 47).

The induction of germinal center reaction and the antigen-specific T cell effector function could only be detected in dLN or in the spleen, while the corresponding gene networks were not detected in whole blood. In fact, the transcriptome perturbation detected in peripheral blood is thought to reflect the overall response of the immune system to vaccine stimulation, even if it is not possible to detect finely tuned responses or phenomena localized in specific lymph nodes.

Taken together, our data suggest that the reprogramming of the recall immune response by the adjuvant used for priming impinges on both innate and adaptive arms resulting, upon boosting, in a stronger recall innate response essential for shaping the downstream adaptive response.

## Ethics Statement

This study was carried out in accordance with the recommendations of Italian national guidelines (Decreto Legislativo 26/2014). The protocol was approved by the Italian Ministry of Health with authorization no. 1004/2015-PR on September 22 2015.

## Author Contributions

EP, DK, FS, AC, GP, and DM conceived and designed the experiments; EP, FF, and FS performed the experiments; GG and IE performed library preparation and sequencing; EP, DK, FS, FF, AC, GP, and DM analyzed the data; FS, EP, DM, and DK: wrote the paper; PA: provided reagents. All the authors read, critically revised, and approved the final manuscript.

## Conflict of Interest Statement

PA is co-inventors on patent applications covering CAF01. As employee, PA has assigned all rights to Statens Serum Institut, a Danish non-profit governmental institute.

## Footnotes

^1^https://reactome.org/ (Accessed: November 20, 2017).
